# Supplementary material for: Discovery of the most compact 3+1-type quadruple star system TIC 120362137
Source: Nat Commun. 2026 Mar 3;17:1859. doi: 10.1038/s41467-026-69223-4 (PMC12957322; doi:10.1038/s41467-026-69223-4)
Supplement: Supplementary file 1 — Supplementary Information [file 41467_2026_69223_MOESM1_ESM.pdf]

Discovery of the most compact 3+1-type quadruple star system TIC 120362137

T. Borkovits, S. A. Rappaport, H.-L. Chen, G. Torres, T. Mitnyan, V. B. Kostov, B. P. Powell, T. Pribulla, P. Zasche, I. B. Bíró, I. Csányi, D. R. Czavalinga, Z. Dencs, J. Kolář, P. Cagaš, Z. Henzl, T. Kaye, H. Kučáková, M. Mašek, R. Uhlař

Supplementary Discussion

The future evolution of this system has also been explored using the stellar evolution code Modules for Experiments in Stellar Astrophysics (MESA, version 12115[1-5]). Naturally, in such an exercise we must make certain reasonable assumptions, and we recognize that any such projections into the future carry with them a degree of uncertainty. However, overall we have confidence in the basics of these evolution calculations. We assume the stars in the system have a metallicity of $Z=0.02$ (with a hydrogen abundance, $X=0.70$, and a helium abundance, $Y=0.28$). In addition, we adopt a mixing-length parameter of $\alpha = l/H_p = 2.0$ and ignore overshooting in the model, which has only a minor effect on our results. We construct the initial stellar model of each component in the system by evolving a zero-age main-sequence star of the same mass until its luminosity approaches the observed value. We make use of the evolve_both_stars and star_plus_point_mass test suites for our binary evolution modelling. In the following, we define the current stage of the system as $t=0$ (first row of Supplementary Figure 1). When the primary in binary A, star Aa, evolves to fill its Roche lobe with a radius of 5.4 $R_\odot$, it has a He core mass of 0.21 $M_\odot$ and starts mass transfer to star Ab (this stage is called as `Roche lobe overflow'; RLOF). At this point, $t = 182.6$ Myr, star Aa is starting to ascend the RGB and star Ab is on the main sequence (second row of Supplementary Figure 1).

From our model, we find that the mass transfer rates exceed 10^{-4} and even 10^{-3} $M_\odot$/yr. The accretor fills its Roche lobe at the same time. Because the donor star (Aa) is more massive than the accretor (Ab), their mass ratio being 1.28, and the outer envelope of the donor star is convective, the subsequent mass transfer is dynamically unstable. Therefore, the binary system will enter a common envelope (CE) phase.

Adopting the $\alpha_{\rm CE}$ prescription (i.e., energy formalism[6,7]) for the CE evolution, and taking $\alpha_{\rm CE}$= 1.0, we find that the CE cannot be ejected and the binary system will merge into a single star, star A'. Given that star Aa has a substantial He core while star Ab is still a MS star, the merged star A' is then a single stellar object ascending the RGB. The mass loss during the merger is quite uncertain, but we adopt the proposition that very little mass can be ejected from the system because there is insufficient gravitational potential energy release to eject it. We take the merger to be conservative, but if, e.g., 10-30% of the total mass were ejected that would not qualitatively alter the subsequent evolution steps.

To construct a model for the merged star A', we simulate the evolution of 1.748 $M_\odot$ RG star with a He core mass of 0.21 $M_\odot$ that is accreting matter at a rate of $10^{-3} M_\odot$/yr until the accreted mass reaches the mass of the star Ab (i.e., an additional 1.361 $M_\odot$). Here we simply assume that the chemical composition of the accreted matter is the same as that of the surface of star Aa (third row of Supplementary Figure 1).

As the merged star A' (of total mass 3.1 $M_\odot$) evolves, it will fill its Roche lobe when its core mass is 0.34$M_\odot$ and it attains a radius of 33.46 $R_\odot$. It will then start mass transfer onto star B. At this time, t = 275.6 Myr, star B is also evolving and has developed a core mass of 0.146 $M_\odot$ (fourth row of Supplementary Figure 1). The mass transfer in this system is also dynamically unstable, and the system will enter a second CE phase. Again, assuming a CE ejection efficiency of 1.0, we find that the CE cannot be ejected, and the system will merge into a single star, star AB (bottom row of Supplementary Figure 1).

At this point we should note that we assume neither of the two mergers leads to the dissolution of the remaining triple, and later, binary systems. Considering the fact that such a merger, in general, is not a violent event (in contrast to a supernova-kick which may lead to catastrophic consequences for the stability of any remaining binary or multiple system) and, moreover, the remaining systems are wide enough, such assumptions appear to be quite realistic.

In our simulation, we assume that the new star AB has a mass of 4.59 $M_\odot$ with a core mass of 0.486 $M_\odot$, i.e. the combined core and total mass of star A' and B. The new star AB starts to transfer material to star C when its core mass is 0.823 $M_\odot$ and it attains a radius of nearly 288 $R_\odot$. At this point, t = 304.2 Myr, star C is still on the MS (first row of Supplementary Figure 2). Given the long orbital period, the system can survive the ensuing CE (second row of Supplementary Figure 2), producing a binary system consisting of a ~0.82 $M_\odot$ CO white dwarf (WD) and ~1 $M_\odot$ MS companion. The binary orbital period after the CE will be ~5.43, or ~27.79 days if we adopt an ejection efficiency of 0.25, or 1.0 (third row of Supplementary Figure 2).

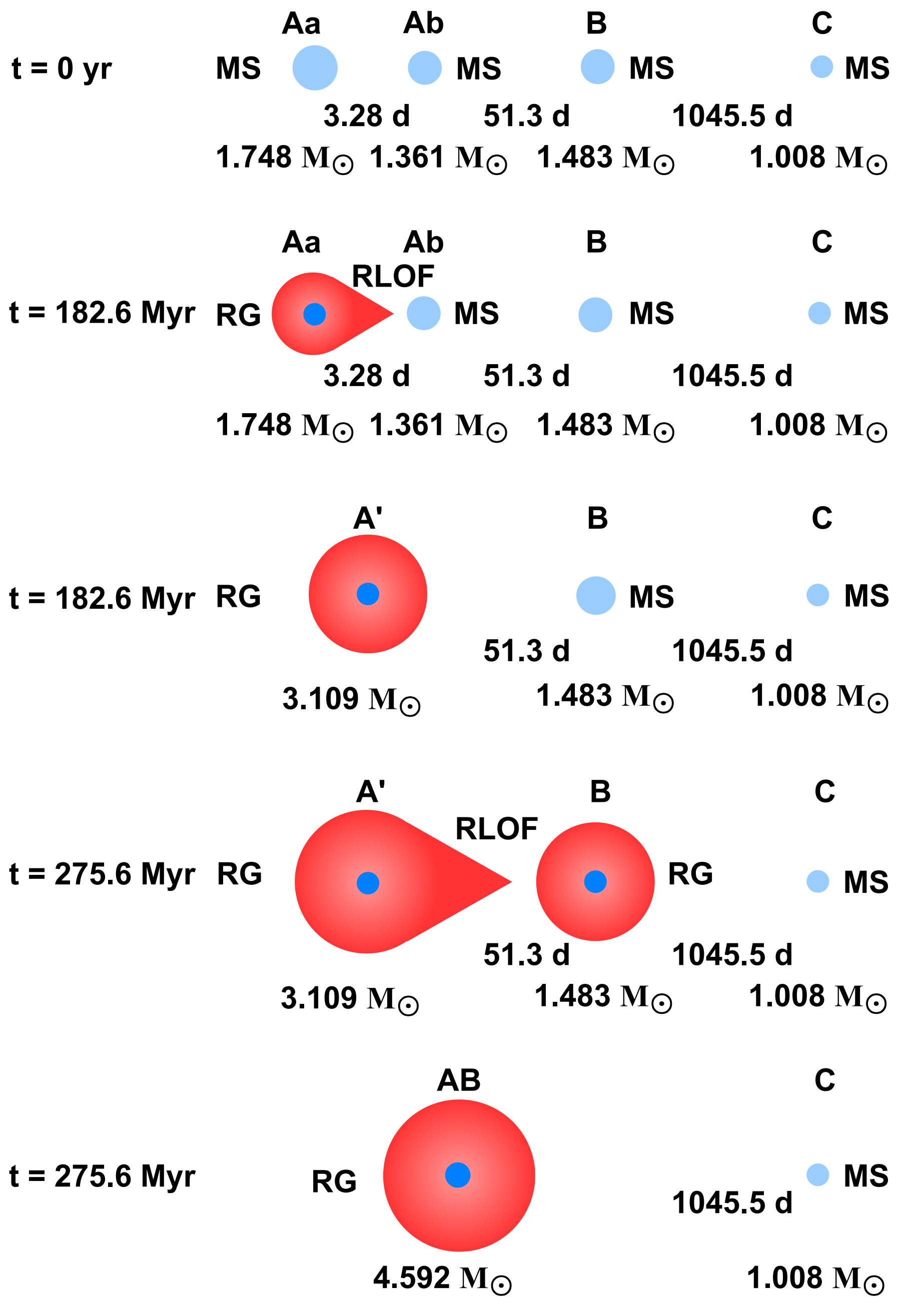

Supplementary Figure 1: The future evolution of the components of TIC 120362137, from the present to the stage of a wide red giant (RG) - main sequence (MS) star binary. The first column show the time counted from the current epoch. Letters Aa, Ab, B, C, A' and AB represent the actual stellar components as they were described in the text. Light blue disks stand for MS components, while larger, red disks with blue cores denote RG stars. In the second and fourth row the drop-like shape of the RG components show the effect of the filling of the current (drop-like shape) Roche lobes, which results in Roche lobe overflow (RLOF). The orbital periods of each binary or triple subsystems are given in days in between the corresponding stellar components, while the actual masses of the stellar components are given in solar masses. Further details can be read in the text.

Finally, star C will start mass transfer on the RGB stably to the CO WD (fourth row of Supplementary Figure 2), eventually leaving a compact double WD system (bottom row of Supplementary Figure 2). Here we take the CO WD as a point mass and do not follow its structure evolution in the binary evolution modelling. In addition, we assume that only 10% of the transferred material can be retained by the WD and other material will leave the system taking away the specific angular momentum of the WD. The properties of the double WD produced from such a quadruple system is very similar to that produced from isolated binaries.
It is interesting to note that if such a double WD system is found today, the observers would likely have no idea that it might have come from such an exotic compact 3+1 quadruple system with an outer period of about a thousand days.

It is worth noting that we adopted a conservative mass transfer for binary Aa-Ab and A'-B. However, as noted above, the mass transfer in these processes should not be fully conservative. This will not change the overall evolution picture. However, the masses of star A' and AB will be different than depicted in Supplementary Figure 1, and the final WD mass and orbital period should be also somewhat different. In addition, we did not consider the effects of rotation on the current simulation. Given that the rotation velocities of these stars are not large, this will not alter the overall evolution scenario, but the WD masses will be slightly larger due to the effect of rotation.

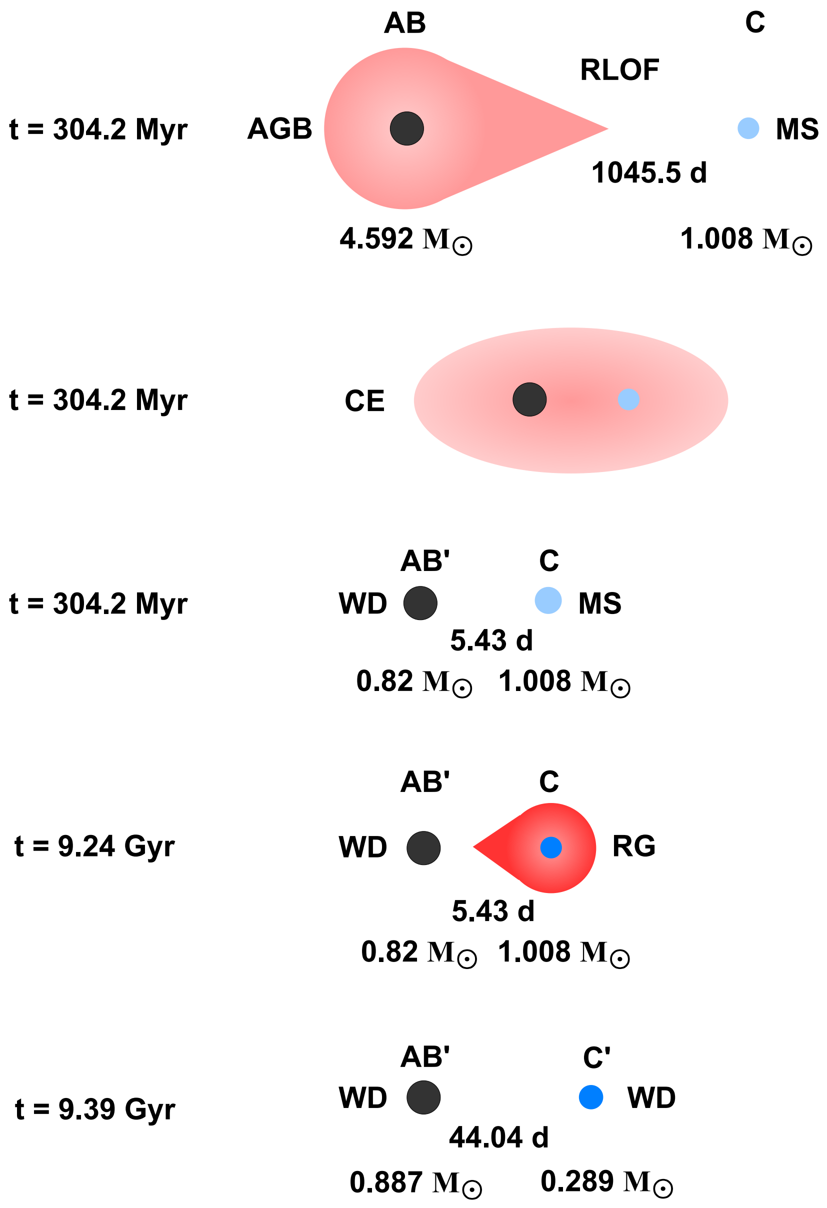

Supplementary Figure 2: The future evolution of the components of TIC 120362137, from the stage of a wide asymptotic giant branch (AGB) - main sequence (MS) star binary towards its final, double white dwarf (WD) state. The first column show the time counted from the current epoch. Letters AB, AB', C and C' represent the actual stellar components as they were described in the text. Light blue disk stand for MS component, while larger, light red disks with black cores denote an AGB star and its non-spherical atmosphere. Finally, black disks stand for WD stars. In the first and fourth rows the drop-like shapes of the AGB and RG components show the effect of filling the current Roche lobes, which results in Roche lobe overflow (RLOF), while in the second row the ellipsoidal shape with two cores stands for a common envelope (CE) phase, when both stellar cores revolve within one stellar atmosphere. The orbital periods of each binary subsystem are given in days in between the corresponding stellar components, while the actual masses of each stellar component is given in solar masses. Further details can be read in the text.

Additional Supplementary Figures and Tables

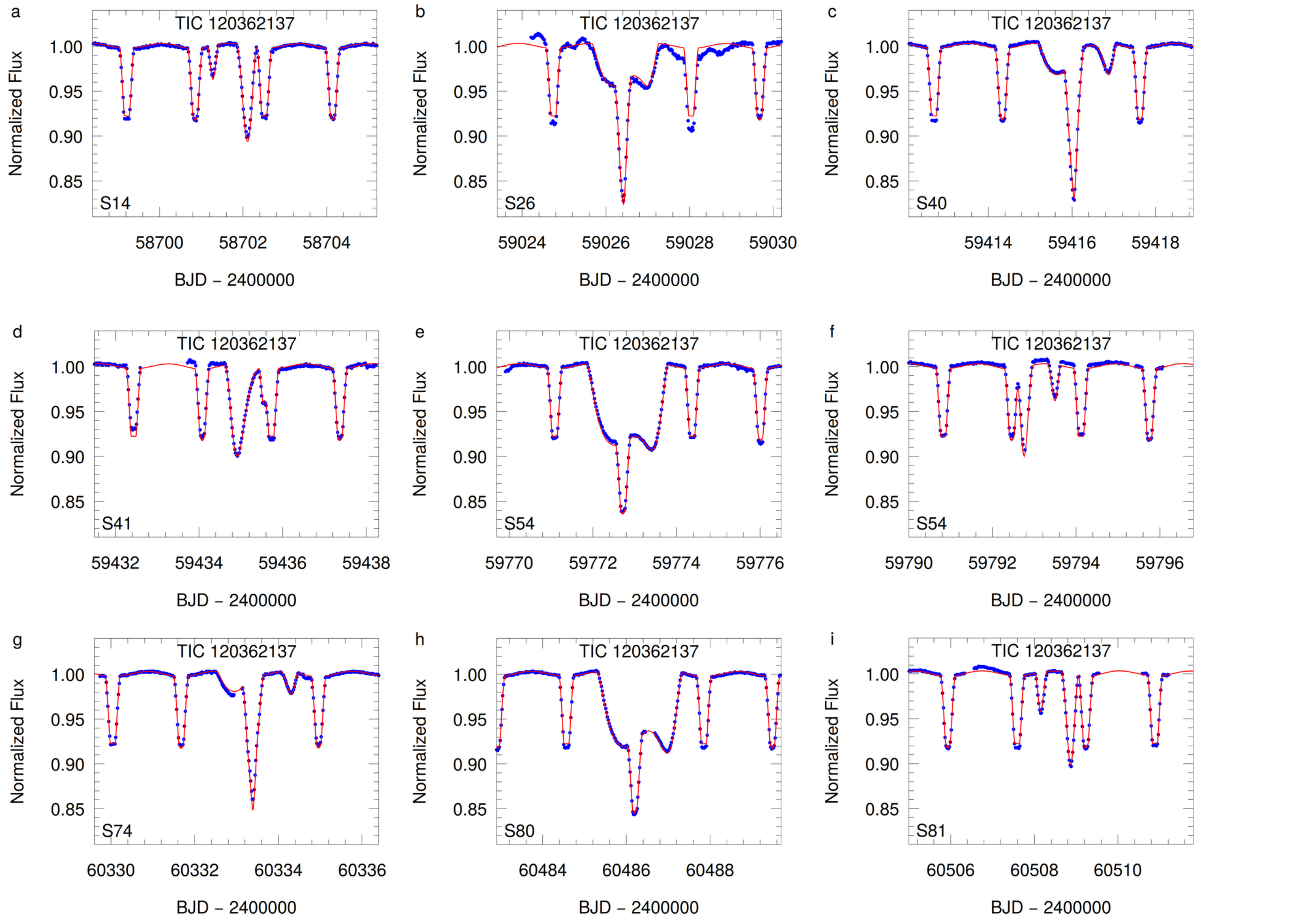

Supplementary Figure 3: TESS-observed third-body eclipses of TIC 120362137. a-i The observed light curve data are plotted with blue points, while the smooth, red curves represents the light curve emulated from the best-fitted (that is, lowest χ^2 value) complex photodynamical solution. The sector numbers are indicated in the lower left corner of each panel. The TESS light curves are binned to 1800 sec averages independent of the original cadence times. Calculation of the model curves is discussed in Methods subsection Spectro-photodynamical analysis subsection of the main text. Source data are provided as a Source Data file.

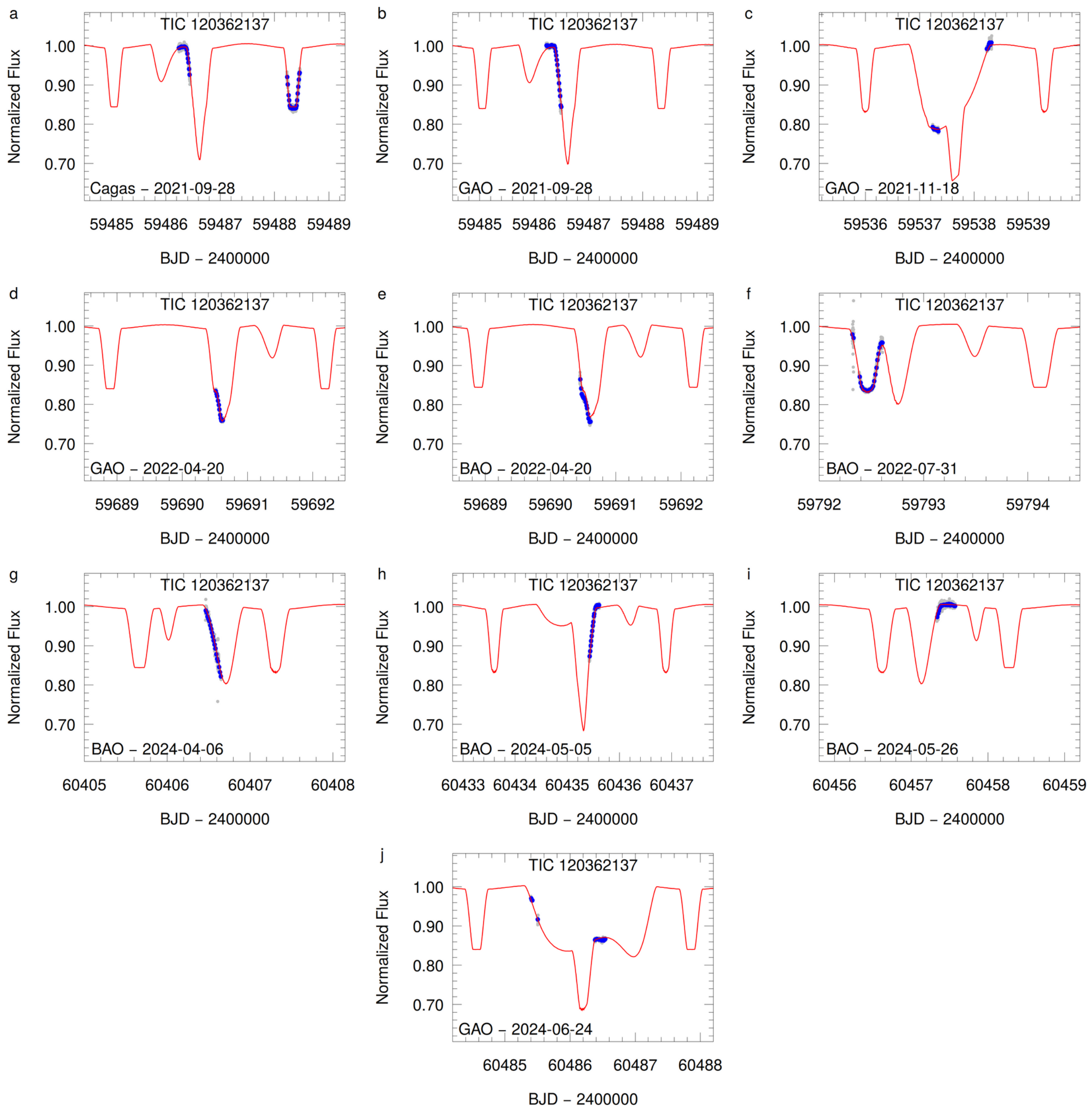

Supplementary Figure 4: Partially observed third-body eclipses of TIC 120362137 from different ground-based observatories and observers. a-j. The dates of the observations, as well as the acronyms of the observational sites are given in the left corners of each panel. Gray dots represent the individual observations, while larger blue circles denote their 15-min. averages which were used for the joint spectro-photodynamical analysis. Red curves represent the best-fit photodynamical model. Source data are provided as a Source Data file.

Location	Instrument	First/last observations	Nights	Notes
Gothard Astr. Obs., Szombathely, H	80-cm RC (GAO80)	03 2021 - 09 2024	22	Sloan *z*'-band, see[8]
Baja Astr. Obs., Baja, H	80-cm RC (BAO80)	06 2021 - 05 2024	10	Sloan *r*'-band, see[8]
Ondřejov Obs., Cz	65-cm	05 2021 - 05 2022	5	Cousins R_C-filter, see[9]
Veltěže u Loun, Cz	20.3-cm and 15-cm	04 2021 - 09 2021	9	Cousins R_C-filter
Jílové u Prahy, Cz	20-cm and 15-cm	04 2021 - 07 2024	8	Cousins R_C-filter
Patterson Obs., AZ, USA	RC50-cm	06 2021	2	Unfiltered, see[8]
BS OBs., Zlin, Cz	30-cm	09 2021	2	https://www.tcmt.org/index.html
Ždánice Obs., Cz	80-cm RC+C5A-150M cam	09 2024	1	Sloan *r*'-band, see https://astro.physics.muni.cz/observatory/

Supplementary Table 1: Log of photometric follow-up observations. The photometric light curves obtained during these observations are provided as Suplementary Data 2-5. Moreover, mid-eclipse times determined from these photometric observations are tabulated in Supplementary Table S3 and provided as machine readable plain ascii data in Supplementary Data 6.

Location	Instrument	First/last observation	Nights	Resolution
Fred Whipple Obs., AZ, USA	1.5m + TRES	09 2020 - 11 2024	73	44000
Skalnaté Pleso Obs., SK	NC130+MUSICOS-clone	06 2021 - 10 2024	20	38000
Piszkéstető, H	RCC100+échelle	03 2021 - 08 2024	26	20000
Rozhen Obs., Bg	RCC200+échelle	05 2022 - 09 2022	2	30000

Supplementary Table 2: Log of spectroscopic follow-up observations. Each individual spectrum obtained during these observations can be downloaded from Zenodo site using the following URL: https://zenodo.org/records/17914597 . Moreover, all the RV data obtained from these measurements are tabulated in Supplementary Tables S4-S6 and provided as machine readable plain ascii data in Supplementary Data 7-9.

BJD -2400000	Cycle no.	Std. Dev. (d)	BJD -2400000	Cycle no.	Std. Dev. (d)	BJD -2400000	Cycle no.	Std. Dev. (d)	BJD -2400000	Cycle no.	Std. Dev. (d)
58684.42478	0.0	0.00006	59381.46942	212.0	0.00003	59662.58453	297.5	0.00009	60331.67572	501.0	0.00006
58686.07944	0.5	0.00006	59391.34050	215.0	0.00005	59718.48141	314.5	0.00004	60336.61238	502.5	0.00006
58687.70980	1.0	0.00008	59392.99104	215.5	0.00005	59744.79968	322.5	0.00006	60338.24874	503.0	0.00004
58689.36348	1.5	0.00009	59394.62565	216.0	0.00006	59746.43521	323.0	0.00007	60410.59481	525.0	0.00013
58690.99543	2.0	0.00008	59396.27748	216.5	0.00007	59751.37444	324.5	0.00006	60415.53568	526.5	0.00006
58692.64860	2.5	0.00006	59397.91137	217.0	0.00006	59753.00812	325.0	0.00007	60443.46381	535.0	0.00008
58694.28220	3.0	0.00009	59399.56274	217.5	0.00005	59754.66001	325.5	0.00007	60481.28393	546.5	0.00005
58695.93412	3.5	0.00008	59401.19611	218.0	0.00008	59757.94511	326.5	0.00005	60482.91957	547.0	0.00005
58697.56730	4.0	0.00011	59402.84734	218.5	0.00007	59759.57869	327.0	0.00006	60484.56905	547.5	0.00005
58699.22163	4.5	0.00007	59406.13252	219.5	0.00005	59761.22906	327.5	0.00005	60486.20099	548.0	0.00007
58700.85526	5.0	0.00007	59407.76642	220.0	0.00006	59764.51543	328.5	0.00005	60489.49196	549.0	0.00005
58702.51037	5.5	0.00011	59409.41732	220.5	0.00005	59766.14965	329.0	0.00005	60491.14126	549.5	0.00004
58704.14399	6.0	0.00010	59411.05245	221.0	0.00006	59767.80114	329.5	0.00005	60492.77979	550.0	0.00006
58705.79954	6.5	0.00007	59412.70359	221.5	0.00005	59771.08763	330.5	0.00007	60494.42834	550.5	0.00004
58707.43620	7.0	0.00009	59414.33876	222.0	0.00005	59774.37398	331.5	0.00011	60496.06736	551.0	0.00004
58709.09124	7.5	0.00010	59417.62643	223.0	0.00006	59776.00832	332.0	0.00011	60497.71648	551.5	0.00005
59011.58647	99.5	0.00014	59420.91455	224.0	0.00012	59779.29864	333.0	0.00008	60499.35531	552.0	0.00004
59013.21585	100.0	0.00017	59422.56613	224.5	0.00022	59780.95039	333.5	0.00010	60501.00587	552.5	0.00004
59014.87131	100.5	0.00015	59424.20524	225.0	0.00022	59784.24101	334.5	0.00009	60502.64341	553.0	0.00005
59016.50923	101.0	0.00011	59425.85706	225.5	0.00019	59785.87679	335.0	0.00008	60504.29440	553.5	0.00005
59018.16118	101.5	0.00013	59427.49415	226.0	0.00014	59787.52880	335.5	0.00008	60505.93130	554.0	0.00018
59019.79719	102.0	0.00017	59429.14576	226.5	0.00013	59789.16718	336.0	0.00009	60507.58963	554.5	0.00004
59021.45067	102.5	0.00016	59430.78388	227.0	0.00012	59790.82285	336.5	0.00006	60509.23151	555.0	0.00004
59024.74548	103.5	0.00012	59432.43711	227.5	0.00013	59794.11772	337.5	0.00010	60510.88439	555.5	0.00006
59028.03712	104.5	0.00014	59434.07940	228.0	0.00009	59795.75430	338.0	0.00009	60512.52352	556.0	0.00008
59029.67348	105.0	0.00013	59435.73115	228.5	0.00010	60111.39727	434.0	0.00014	60514.17381	556.5	0.00007
59031.32647	105.5	0.00013	59437.37059	229.0	0.00011	60313.61292	495.5	0.00004	60515.81027	557.0	0.00004
59032.96019	106.0	0.00013	59439.02347	229.5	0.00012	60315.24708	496.0	0.00005	60517.46127	557.5	0.00006
59034.61202	106.5	0.00011	59440.65995	230.0	0.00010	60316.89883	496.5	0.00006	60519.09676	558.0	0.00010
59325.56984	195.0	0.00016	59442.31111	230.5	0.00009	60318.53322	497.0	0.00009	60520.74708	558.5	0.00004
59330.51296	196.5	0.00008	59443.94598	231.0	0.00010	60320.18431	497.5	0.00006	60522.38212	559.0	0.00004
59340.38499	199.5	0.00033	59445.59733	231.5	0.00012	60321.81821	498.0	0.00005	60524.03280	559.5	0.00004
59353.52571	203.5	0.00014	59460.37465	236.0	0.00009	60323.46956	498.5	0.00005	60525.66791	560.0	0.00005
59358.44170	205.0	0.00014	59465.30783	237.5	0.00019	60325.10366	499.0	0.00006	60527.31782	560.5	0.00004
59363.38081	206.5	0.00035	59488.34223	244.5	0.00004	60326.75482	499.5	0.00003	60528.95353	561.0	0.00005
59376.53863	210.5	0.00018	59516.26245	253.0	0.00010	60328.38949	500.0	0.00005	60530.60367	561.5	0.00004
59379.82814	211.5	0.00008	59634.63015	289.0	0.00010	60330.03986	500.5	0.00007	60532.23918	562.0	0.00005

Supplementary Table 3: Eclipse times of the inner EB of TIC 120362137. Columns #1, #4, #7 and #10 give the derived mid-minimum moments in modified barycentric Julian date (i. e., BJD - 2400000). Columns #2, #5, #8 and #11 tabulate the current cycle numbers. Integer and half-integer cycle numbers denote primary and secondary eclipses, respectively. Furthermore, columns #3, #6, #9, and #12 gives the standard deviations of each mid-minimum moment calculations in days. This Table is provided also in a machine readable, plain ascii format in the Supplementary Data section.

BJD -2400000	RV_{Aa} kms^{-1}	σ_{Aa} kms^{-1}	RV_{Ab} kms^{-1}	σ_{Ab} kms^{-1}	RV_{B} kms^{-1}	σ_{B} kms^{-1}	RV_{C} kms^{-1}	σ_{C} kms^{-1}
59103.6624	-22.55	2.57	...	...	-2.07	14.38	-20.04	3.74
59111.6187	31.56	2.45	-60.36	2.95	-44.20	13.74	-14.95	3.58
59116.7021	-12.48	2.50	26.50	3.00	-86.80	13.99	-23.05	3.64
59472.7149	92.10	1.58	-118.51	1.90	-76.34	8.87	-7.78	2.31
59486.6802	...	...	-12.04	3.05	-41.36	14.19	-8.99	3.69
59500.6531	-142.17	1.58	69.65	1.90	23.19	8.86	-8.51	2.30
59522.6263	34.05	1.63	-49.33	1.96	-61.27	9.12	-11.20	2.37
59529.6052	-17.13	1.42	54.41	1.70	-106.53	7.94	-9.95	2.06
59531.5894	87.93	1.63	-88.89	1.96	-86.50	9.12	-13.89	2.37
59650.9995	...	...	-69.70	1.76	36.47	8.21	-28.35	2.13
59660.9527	...	...	...	...	18.15	8.24	-30.45	2.14
59676.9545	70.80	1.68	-79.68	2.02	-84.10	9.42	-30.91	2.45
59681.0012	-40.66	1.82	87.22	2.18	-87.57	10.18	-28.35	2.65
59691.8922	-67.68	1.38	42.67	1.66	-26.56	7.74	-31.02	2.01
59696.9936	-15.65	1.79	-65.29	2.15	20.18	10.02	-29.87	2.61
59700.9839	-128.65	1.61	58.76	1.94	25.49	9.02	-30.39	2.35
59713.8439	-84.94	1.42	30.26	1.71	-0.45	7.98	-32.85	2.08
59722.8937	66.65	1.31	-109.52	1.58	-39.76	7.34	-34.89	1.91
59732.9093	76.91	1.09	-58.77	1.31	-94.95	6.12	-39.72	1.59
59743.9122	-116.47	1.28	91.69	1.54	-24.41	7.20	-34.73	1.87
59767.7986	-22.14	1.51	...	...	3.97	8.48	...	...
59820.7782	38.07	1.52	-99.92	1.83	1.77	8.54	-47.89	2.22
59837.6786	111.01	1.07	-99.60	1.29	-83.58	6.00	-45.48	1.56
59839.6987	-55.32	1.33	100.43	1.60	-77.94	7.44	-43.04	1.94
59842.7807	-89.02	1.38	107.28	1.66	-51.46	7.71	-49.28	2.01
59850.6226	45.86	1.33	-148.81	1.59	33.58	7.44	-48.78	1.93
59861.6697	-70.94	1.62	-7.00	1.95	21.52	9.07	-43.49	2.36
59878.6510	-88.23	1.68	117.25	2.01	-55.74	9.39	-47.73	2.44
59880.6189	92.38	1.51	-99.69	1.81	-66.40	8.46	-53.56	2.20
59903.5745	46.33	1.50	-154.31	1.81	46.12	8.42	-48.65	2.19
60035.0039	102.57	1.52	-106.10	1.83	-68.01	8.52	-47.04	2.22
60041.9530	81.33	1.30	-61.48	1.56	-88.54	7.28	-49.08	1.89
60050.9488	34.21	1.46	-104.71	1.75	11.29	8.17	-45.50	2.13
60070.8988	50.84	1.21	-140.77	1.46	19.85	6.79	-39.66	1.77
60090.9682	106.71	1.23	-95.46	1.47	-86.59	6.87	-36.99	1.79
60091.8098	-9.60	1.44	49.62	1.73	-106.12	8.09	-39.88	2.10
60097.8633	58.41	0.95	-83.43	1.14	-44.68	5.32	-38.99	1.38
60102.7767	-95.98	1.58	50.05	1.90	-2.31	8.83	-33.83	2.30
60108.8113	-135.88	1.25	70.13	1.51	29.93	7.02	-33.69	1.83
60114.8385	-74.78	1.97	-12.61	2.36	23.65	11.00	-34.37	2.86
60115.8091	-122.45	1.35	54.47	1.63	16.90	7.59	-29.97	1.97
60126.6973	49.99	1.40	-121.95	1.68	4.06	7.83	-27.28	2.04
60129.8240	39.25	1.53	-90.82	1.84	-14.31	8.57	-24.94	2.23
60144.7515	-61.78	1.59	106.17	1.91	-81.42	8.89	-24.42	2.31
60151.7427	-114.19	1.15	92.86	1.39	-28.35	6.46	-18.56	1.68
60159.6546	31.20	1.34	-154.83	1.61	35.30	7.49	-13.76	1.95
60161.7952	-132.61	1.46	51.00	1.76	31.19	8.21	-15.80	2.13
60162.7223	5.98	1.61	-126.50	1.93	33.67	9.01	-17.18	2.34
60204.6169	-112.44	1.28	56.36	1.54	-15.28	7.18	-2.12	1.87
60207.6599	-139.19	0.92	69.50	1.11	8.37	5.18	-0.36	1.35
60215.6432	33.99	1.43	-166.16	1.72	33.46	8.00	0.95	2.08
60227.6626	-103.28	1.14	54.65	1.38	-22.34	6.41	5.07	1.67
60235.6909	76.69	1.31	-122.74	1.57	-57.43	7.32	6.61	1.90
60245.5850	92.37	1.52	-102.43	1.83	-93.22	8.52	9.45	2.22
60256.5829	-122.21	1.30	56.59	1.57	-7.50	7.29	8.42	1.90
60406.0034	29.94	0.96	-90.56	1.16	...	...	6.80	1.41
60422.9131	38.70	1.40	-168.67	1.68	30.79	7.81	2.46	2.03
60430.9888	-125.09	1.17	76.24	1.41	-25.40	6.55	4.05	1.71
60445.8361	95.42	1.38	-111.29	1.66	-85.31	7.76	2.63	2.02
60452.8857	71.88	1.26	-88.37	1.52	-88.33	7.07	-1.20	1.84
60458.9501	54.28	1.40	-145.82	1.69	-5.95	7.86	1.18	2.05
60478.8878	49.21	1.46	-158.40	1.75	12.15	8.15	-4.93	2.12
60493.8220	-84.93	1.38	104.34	1.66	-79.07	7.71	-8.45	2.01
60504.7163	66.07	1.57	-87.24	1.89	-75.03	8.81	-3.39	2.29
60509.6985	-101.24	1.74	64.54	2.10	-25.42	9.78	-7.20	2.54
60522.7367	-111.87	1.43	22.44	1.72	22.85	8.03	-8.50	2.09
60555.6436	-60.38	1.03	81.01	1.24	-75.60	5.76	...	...
60566.6397	...	...	-19.78	1.43	21.55	6.68	-11.78	1.74
60577.6328	42.83	0.84	-156.53	1.01	20.43	4.71	-12.21	1.22
60588.6687	-98.72	1.28	81.81	1.54	...	...	-13.74	1.86
60628.5531	-132.98	1.28	71.70	1.54	7.90	7.17	-20.37	1.86
60633.5708	51.97	1.35	-140.80	1.62	1.30	7.55	-20.66	1.96
60643.5727	64.61	1.42	-99.36	1.71	-54.50	7.98	-21.62	2.08

Supplementary Table 4: TRES spectrograph RV measurements of TIC 120362137. BJD-2400000 represents the mid-time of the given spectroscopic observation in modified barycentric Julian date. $RV_{Aa,Ab,B,C}$ stand the RV of the denoted component, while $\sigma_{Aa,Ab,B,C}$ represent the corresponding uncertainties of any given measurements. This Table is provided also in a machine readable, plain ascii format in the Supplementary Data section.

BJD -2400000	RV_{Aa} kms^{-1}	σ_{Aa} kms^{-1}	RV_{Ab} kms^{-1}	σ_{Ab} kms^{-1}	RV_B kms^{-1}	σ_B kms^{-1}	RV_C kms^{-1}	σ_C kms^{-1}
59372.54510	-84.19	0.40	119.91	0.72	...	...	...	...
59383.47483	38.75	1.12	-91.05	1.06	...	...	7.36	0.98
59385.47961	-115.38	0.65	89.01	1.00	...	...	2.26	1.13
59386.52045	-17.65	1.35	-68.83	0.97	...	...	8.91	0.69
59388.44509	-102.99	0.35	41.73	0.43	...	...	3.36	0.40
59389.49576	-72.70	0.86	2.78	0.89	...	...	5.11	1.35
59392.42470	-130.38	0.82	52.48	1.08	...	...	2.72	1.51
59403.50579	37.43	0.72	-163.00	0.92	...	...	3.41	0.70
59403.54015	34.21	0.98	-164.84	1.11	...	...	3.64	1.06
59649.61084	-27.14	2.93	-89.37	2.23	...	...	...	...
59659.61201	...	...	-110.71	1.74	...	...	-25.45	0.94
59715.45370	13.67	1.72	-89.17	2.53	...	...	-32.89	1.42
60585.27921	-93.01	0.88	58.26	1.75	...	...	-11.21	1.72
60600.27480	97.33	1.05	-100.70	1.56	...	...	-18.32	1.92
60603.28366	72.44	1.08	-62.49	1.09	...	...	-17.76	0.93

Supplementary Table 5: Skalnaté Pleso Observatory RV measurements of TIC 120362137. BJD-2400000 represents the mid-time of the given spectroscopic observation in modified barycentric Julian date. $RV_{Aa,Ab,B,C}$ stand the RV of the denoted component, while $\sigma_{Aa,Ab,B,C}$ represent the corresponding uncertainties of any given measurements. This Table is provided also in a machine readable, plain ascii format in the Supplementary Data section.

BJD -2400000	RV_{Aa} kms^{-1}	σ_{Aa} kms^{-1}	RV_{Ab} kms^{-1}	σ_{Ab} kms^{-1}	RV_B kms^{-1}	σ_B kms^{-1}	RV_C kms^{-1}	σ_C kms^{-1}
59294.58282	-19.16	7.40	-103.02	7.18	...	...	1.04	6.78
59296.59052	-136.09	16.29	51.26	15.70	...	...	...	...
59298.59868	45.73	1.55	-166.34	4.47	...	...	...	...
59328.48773	49.76	9.15	-52.67	5.90	...	...	...	...
59329.46319	-84.06	5.72	...	...	...	...	...	...
59331.49172	86.31	5.60	...	...	...	...	-4.12	10.21
59332.42748	-79.64	4.41	30.19	6.13	...	...	...	...
59332.46919	-75.29	6.44	40.07	7.57	...	...	...	...
59360.56366	26.33	0.58	-121.92	1.59	...	...	...	...
59361.44948	11.07	1.00	-84.95	2.90	...	...	...	...
59390.45791	58.55	1.63	-165.16	0.71	...	...	...	...
59392.43062	-136.11	1.00	51.07	2.26	...	...	-5.82	3.04
59394.52628	-31.39	11.51	-83.71	1.99	...	...	-1.38	4.48
59395.52528	-142.21	1.74	69.30	1.81	...	...	...	...
59658.66088	-129.69	2.99	57.87	8.78	...	...	-38.69	6.67
59659.66742	17.74	6.16	...	...	...	...	-32.62	4.76
59660.64429	...	...	-95.57	15.88	...	...	-16.83	2.38
59711.40636[a]	-113.78	1.76	57.24	1.87	...	...	-32.59	2.98
59786.50678	-78.13	3.68	137.80	3.74	...	...	-36.64	1.96
59826.51719	-74.99	3.83	90.49	4.57	...	...	-32.48	4.37
59831.39569[a]	90.93	1.33	-85.40	1.55	...	...	-41.86	1.98
60426.59771	15.73	3.04	-144.60	1.33	...	...	...	...
60429.59897	...	...	-141.26	2.85	...	...	...	...
60430.59866	-95.97	5.49	...	...	...	...	...	...
60431.60086	-91.82	1.54	...	...	...	...	...	...
60467.49259	-146.71	1.75	...	...	...	...	...	...
60552.46651	...	...	104.96	1.17	...	...	...	...
60553.38550	...	...	63.55	2.37	...	...	...	...

Supplementary Table 6: Konkoly (Piszkéstető) and Rozhen Observatory RV measurements of TIC 120362137. BJD-2400000 represents the mid-time of the given spectroscopic observation in modified barycentric Julian date. $RV_{Aa,Ab,B,C}$ stand the RV of the denoted component, while $\sigma_{Aa,Ab,B,C}$ represent the corresponding uncertainties of any given measurements. The two measurements, denoted by the superscript [a] was obtained with the 2-m telescope of Rozhen Observatory (BG). The others were observed in the Piszkéstető Mountain Station of Konkoly Observatory. This Table is provided also in a machine readable, plain ascii format in the Supplementary Data section.

References

[1] Paxton, B. *et al.* Modules for Experiments in Stellar Astrophysics (MESA). *ApJS* **192**, 3 (2011).

[2] Paxton, B. *et al.* Modules for Experiments in Stellar Astrophysics (MESA): Planets, Oscillations, Rotation, and Massive Stars. *ApJS* **208**, 4 (2013).

[3] Paxton, B. *et al.* Modules for Experiments in Stellar Astrophysics (MESA): Binaries, Pulsations, and Explosions. *ApJS* **220**, 15 (2015).

[4] Paxton, B. *et al.* Modules for Experiments in Stellar Astrophysics (MESA): Convective Boundaries, Element Diffusion, and Massive Star Explosions. *ApJS* **234**, 34 (2018).

[5] Paxton, B. *et al.* Modules for Experiments in Stellar Astrophysics (MESA): Pulsating Variable Stars, Rotation, Convective Boundaries, and Energy Conservation. *ApJS* **243**, 10 (2019).

[6] Webbink, R. F. Double white dwarfs as progenitors of R Coronae Borealis stars and type I supernovae. *ApJ* **277**, 355–360 (1984).

[7] de Kool, M. Common Envelope Evolution and Double Cores of Planetary Nebulae. *ApJ* **358**, 189 (1990).

[8] Borkovits, T. *et al.* Triply eclipsing triple stars in the northern TESS fields: TICs 193993801, 388459317, and 52041148. *MNRAS* **510**, 1352–1374 (2022).

[9] Fatka, P. *et al.* Spins and shapes of 11 near-Earth asteroids observed within the NEOROCKS projekt. *A&Ap* **695**, A139 (2025).
