# Supplementary material for: Discovery of the most compact 3+1-type quadruple star system TIC 120362137
Source: Nat Commun. 2026 Mar 3;17:1859. doi: 10.1038/s41467-026-69223-4 (PMC12957322; doi:10.1038/s41467-026-69223-4)
Supplement: Supplementary file 2 — Description of Additional Supplementary Files [file 41467_2026_69223_MOESM2_ESM.pdf]

Description of additional supplementary files

Name: Supplementary Data 1
Description: Those parts of the 1800-sec-binned TESS observational data, which were used for the spectro-photodynamical analysis (see in the Methods section)
The data columns are:
Col. 1: BJD - 2400000[in days]
Col. 2: Normalized (or relative) flux
Col. 3: Observational uncertainties (in normalized flux)

Name: Supplementary Data 2
Description: The original Sloan-z' filtered GAO80 observations of TIC 120362137 (see in the Methods section).
The data columns are:
Col. 1: BJD - 2400000[in days]
Col. 2: Normalized (or relative) flux
Col. 3: Observational uncertainties (in normalized flux)

Name: Supplementary Data 3
Description: The original Sloan-r' filtered BAO80 observations together with the Cousins R observations of other observers of TIC 120362137 (see in the Methods section).
The data columns are:
Col. 1: BJD - 2400000 [in days]
Col. 2: Normalized (or relative) flux
Col. 3: Observational uncertainties (in normalized flux) - where this was not available, the value is zero

Name: Supplementary Data 4
Description: The 900-sec binned Sloan-z' filtered GAO80 observations of TIC 120362137, which were used for the spectro-photodynamical analysis (see in the Methods section).
The data columns are:
Col. 1: BJD - 2400000[in days]
Col. 2: Normalized (or relative) flux
Col. 3: Observational uncertainties (in normalized flux)

Name: Supplementary Data 5
Description: The 900-sec-binned Sloan-r' filtered BAO80 observations together with the Cousins R observations of other observers of TIC 120362137, which were used for the spectro-photodynamical analysis (see in the Methods section).
The data columns are:
Col. 1: BJD - 2400000 [in days]
Col. 2: Normalized (or relative) flux
Col. 3: Observational uncertainties (in normalized flux) - where this was not available, the value is zero

Name: Supplementary Data 6
Description: Times of minima of TIC 120362137. Integer and half-integer cycle numbers refer to primary and secondary eclipses, respectively.
The data columns are:
Col. 1: BJD - 2400000 [in days]
Col. 2: Cycle number
Col. 3: standard deviation [in days]

Name: Supplementary Data 7
Description: TRES Spectrograph Radial Velocity Measurements of TIC 120362137.
Note: missing data is denoted with "..."
The data columns are:
Col. 1: BJD - 2400000 [in days]
Col. 2: RV_Aa [in km/s]
Col. 3: sigma_(RV_Aa) [in km/s]
Col. 4: RV_Ab [in km/s]
Col. 5: sigma_(RV_Ab) [in km/s]
Col. 6: RV_B [in km/s]
Col. 7: sigma_(RV_B) [in km/s]
Col. 8: RV_C [in km/s]
Col. 9: sigma_(RV_C) [in km/s]

Name: Supplementary Data 8
Description: Skalnate Pleso Observatory Velocity Measurements of TIC 120362137.
Note: missing data is denoted with "..."
The data columns are:
Col. 1: BJD - 2400000 [in days]
Col. 2: RV_Aa [in km/s]
Col. 3: sigma_(RV_Aa) [in km/s]
Col. 4: RV_Ab [in km/s]
Col. 5: sigma_(RV_Ab) [in km/s]
Col. 6: RV_B [in km/s]
Col. 7: sigma_(RV_B) [in km/s]
Col. 8: RV_C [in km/s]
Col. 9: sigma_(RV_C) [in km/s]

Name: Supplementary Data 9
Description: Konkoly and Rozhen Observatory Velocity Measurements of TIC 120362137.
Note: missing data is denoted with "..."
The data columns are:
Col. 1: BJD - 2400000 [in days]
Col. 2: RV_Aa [in km/s]
Col. 3: sigma_(RV_Aa) [in km/s]
Col. 4: RV_Ab [in km/s]
Col. 5: sigma_(RV_Ab) [in km/s]
Col. 6: RV_B [in km/s]
Col. 7: sigma_(RV_B) [in km/s]
Col. 8: RV_C [in km/s]
Col. 9: sigma_(RV_C) [in km/s]
